# Supplementary material for: Longitudinal single-cell profiling of chemotherapy response in acute myeloid leukemia
Source: Nat Commun. 2023 Mar 8;14:1285. doi: 10.1038/s41467-023-36969-0 (PMC9995364; doi:10.1038/s41467-023-36969-0)

Supplementary information for:

Longitudinal single-cell profiling of chemotherapy response in acute myeloid leukemia

Matteo Maria Naldini[1,2], Gabriele Casirati[3,#], Matteo Barcella[1,4,#], Paola Maria Vittoria Rancoita[5], Andrea Cosentino[1,2], Carolina Caserta[1,2], Francesca Pavesi[3], Erika Zonari[1], Giacomo Desantis[1], Diego Gilioli[1,2], Matteo Giovanni Carrabba[3], Luca Vago[3], Massimo Bernardi[3], Raffaella Di Micco[1], Clelia Di Serio[5], Ivan Merelli[4], Monica Volpin[1], Eugenio Montini[1], Fabio Ciceri[3] and Bernhard Gentner[1,3,*]

[#] These authors contributed equally.
[1] San Raffaele Telethon Institute for Gene Therapy (SR-TIGET), IRCCS San Raffaele Scientific Institute, Milan, Italy.
[2] Vita-Salute San Raffaele University, Milan, Italy.
[3] Hematology and Bone Marrow Transplantation Unit, IRCCS San Raffaele Hospital, Milan, Italy.
[4] National Research Council, Institute for Biomedical Technologies, Segrate, Italy
[5] University Center for Statistics in the Biomedical Sciences (CUSSB), Vita-Salute San Raffaele University, Milan, Italy
[*] Corresponding author, lead contact: gentner.bernhard@hsr.it

Supplementary Figure 1

A Flow cytometry gating and sorting strategies for patient sample bone marrow (PB for PT06 at diagnosis) prior to single-cell RNA sequencing. Samples processed with each sorting strategy are reported on the left. Strategy1: Singlets/Physical/CD45$^{+/low}$/Recovery of CD117^{+}34^{-} (Gate CD117^{+}) & CD34^{+} cells. Strategy2: Singlets/Physical/Live/FITC^{-} (CD3^{-}CD19^{-}CD235a^{-})/CD45^{+}/low/ Recovery of CD34^{+} cells; cells not within CD34^{+} gate are furhter divided into recovery gates: CD117^{+} & inverse CD117^{+} gate (NOT-CD117^{+}). Strategy3: Singlets/Physical/CD45$^{+/low}$FITC^{-} (AnnexinV^{-}CD3^{-}CD19^{-}CD235a^{-})/ Recovery of CD34^{+} cells; CD117^{+}34^{-} cells (Gate CD117^{+}); and remaining CD34^{-}117^{-} cells (Gate P4).

B Table summarizing patients' clinical features at diagnosis. UPN: unique patient number; WHO diagnosis: clinical entity according to WHO 2016; ELN 2017: risk stratification according to European Leukemia Net recommendations 2017; Clinical outcome was dichotomized as Favorable, if the patient is alive, and Adverse, if dead at the time of writing; ASCT, autologous stem cell transplantation; HSCT, allogeneic stem cell transplantation; FAB, French-American-British classification; BM blast %, blast count on bone marrow by morphological analysis; for *NPM1*, the mutation type is reported; or *FLT3ITD*, the allelic ratio is reported when available; n.a. not available.

C Table reporting differentially expressed genes (DEGs) between cells harboring the NPM1 mutA transcript (MUT) and those classified as AML by the NPM1-MF algorithm within each cluster identified by unsupervised clustering at a resolution of. 0.6. We identified a non-negligible number of DEGs only for cluster 7, where we observed higher expression of erythroid lineage transcripts within MUT blasts with respect to other AML classified cells (Supplementary Data 2).

D Left: Human engraftment over time in PB of NSG mice transplanted with diagnosis BMMCs of PT01 (black) or PBMCs of PT06 (grey). Right: Human graft composition for PT01 and PT06 at 10 weeks post-transplant. Data are presented as mean +/- SEM. n=6 PDX from 2 patients.

E Left: UMAP plot of the total merged *NPM1*^{mut} AML dataset prior to harmonization process. Right: *NPM1*^{mut} AML dataset post harmonization[1] for correction of patient- and chromium chemistry-derived batch effects. Cells are colored by 10x genomics chromium chemistry used for library production (v2 or v3) or Patient ID.

F Single-cell expression heatmap of the top 10 marker genes of each cluster identified at a resolution of 0.6 for the *NPM1*^{mut} AML dataset. Each column represents a single cell transcriptome and is annotated on top for cluster, patient, and sampling timepoint variables.

G Single-cell expression heatmap of the top 10 marker genes of each cluster identified at a resolution of 0.6 for the del(7) AML dataset. Each column represents a single cell transcriptome and is annotated on top for cluster, patient, and sampling timepoint variables.

Source data are provided as a Source Data file

Supplementary Figure 1:

A

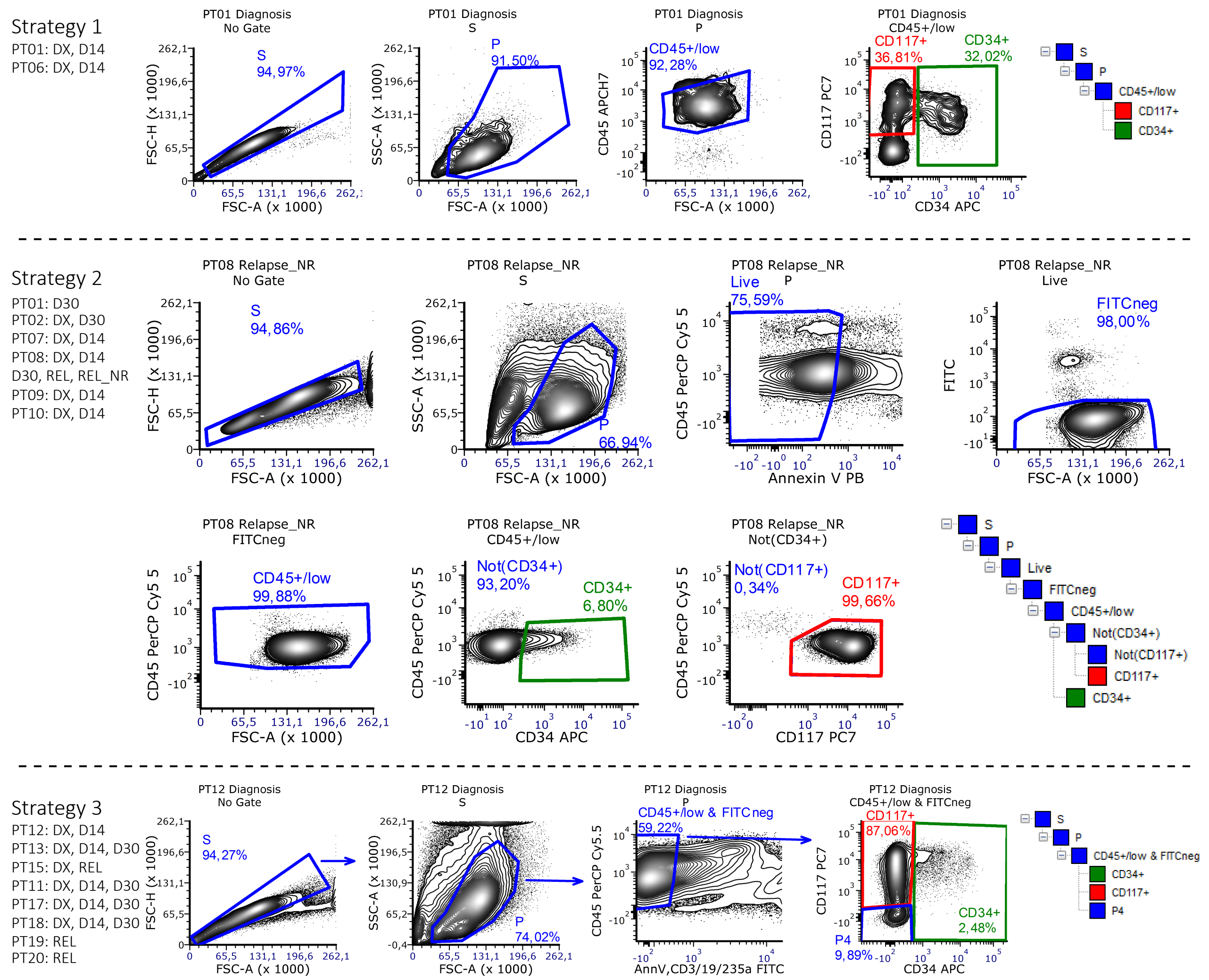

B

UPN	WHO diagnosis	Gender	Age at diagnosis (years)	ELN 2017	Clinical Outcome	Karyotype	FAB	BM blast %	DNMT3A	IDH2	NPM1	FLT3ITD
**PT01**	AML NPM1	M	52	INT	Adverse	46, XY	n.a.	92	n.a.	n.a.	A	0,67
**PT02**	AML NPM1	F	24	INT	Adverse	46, XX	M5a	70	n.a.	n.a.	A	0,77
**PT03**	AML NPM1	F	36	INT	Adverse	46, XX	M2	76	n.a.	n.a.	A	pos
**PT04**	AML NPM1	F	48	n.a.	Favorable	46,XX	M1	86	n.a.	n.a.	A	pos
**PT06**	AML NPM1	F	76	FAV	Favorable	46, XX	M2	75	n.a.	n.a.	A	0,31
**PT07**	AML NPM1	M	42	FAV	Favorable	46, XY	M5	58	n.a.	n.a.	A	neg
**PT08**	AML NPM1	M	52	FAV	Adverse	46, XY	M4	19+14	R882H	R140Q	A	neg
**PT09**	AML NPM1	F	66	INT	Adverse	46, XX	M4	35	n.a.	n.a.	A	5,07
**PT10**	AML NPM1	F	65	FAV	Adverse	na	M5b	21	n.a.	neg	A	neg
**PT12**	AML NPM1	M	44	FAV	Favorable	46, XY	M5a	32	neg	n.a.	A	neg
**PT13**	AML NPM1	M	72	n.a.	Adverse	46, XY, t(10;13)	M2	82	n.a.	n.a.	A	pos
**PT15**	AML NPM1	F	48	FAV	Adverse	46,XX	M5	38	n.a.	n.a.	A	neg
**PT16**	AML NPM1	M	55	INT	Adverse	46,XY, del(10)q?	n.a.	76	n.a.	n.a.	A	0,53
**PT11**	AML DEL(7)	M	19	ADV	Adverse	45, XY -7	M5a	73	n.a.	n.a.	WT	neg
**PT17**	AML DEL(7)	M	33	ADV	Adverse	45, XY -7	M5b	38	n.a.	neg	WT	neg
**PT18**	AML DEL(7)	M	73	ADV	Adverse	45, XY -7	n.a.	42	n.a.	neg	WT	neg
**PT19**	AML NPM1	F	65	INT	Adverse	46, XX	n.a.	n.a.	n.a.	n.a.	A	pos
**PT20**	AML NPM1	F	59	FAV	Favorable	46, XX	n.a.	40	n.a.	n.a.	A	0,052

C

INTRA-Cluster comparison of AML cells harboring the NPM1mut transcript (MUT cells) vs other cells classified as AML by NPM1-MF		
**Cluster**	**# DEGs logFC >0 & p.adj <0.05**	**DEGs:**
0	2	NPM1, RPL31
1	2	NPM1, RPS4Y1
2	4	RPS4Y1, ELANE, SPINK2, RPS10
3	2	NPM1, RPS4Y1
4	8	NPM1, RPL10, RPS3, RPS18, RPS5, RPL10A, RPS10, RPS26
5	1	NPM1
6	1	NPM1
7	224	*See Supplementary Table 2, "Intra-cluster 7 MUT vs Rest"*
8	1	NPM1
9	2	NPM1, REXO2
10	0	
11	2	NPM1, RPL13A
12	0	

DEGs were computed with a logistic regression framework in Seurat to determine differentially expressed genes while controlling for PatientID and Timepoint latent variables.

D

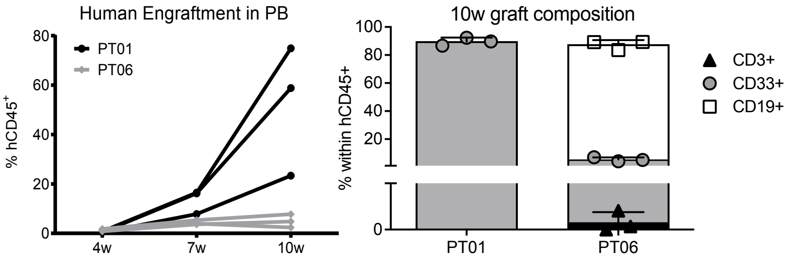

E

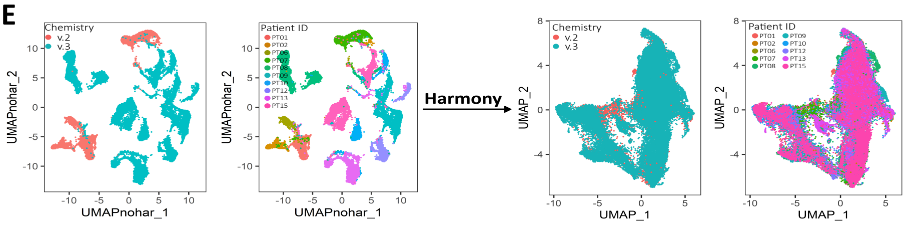

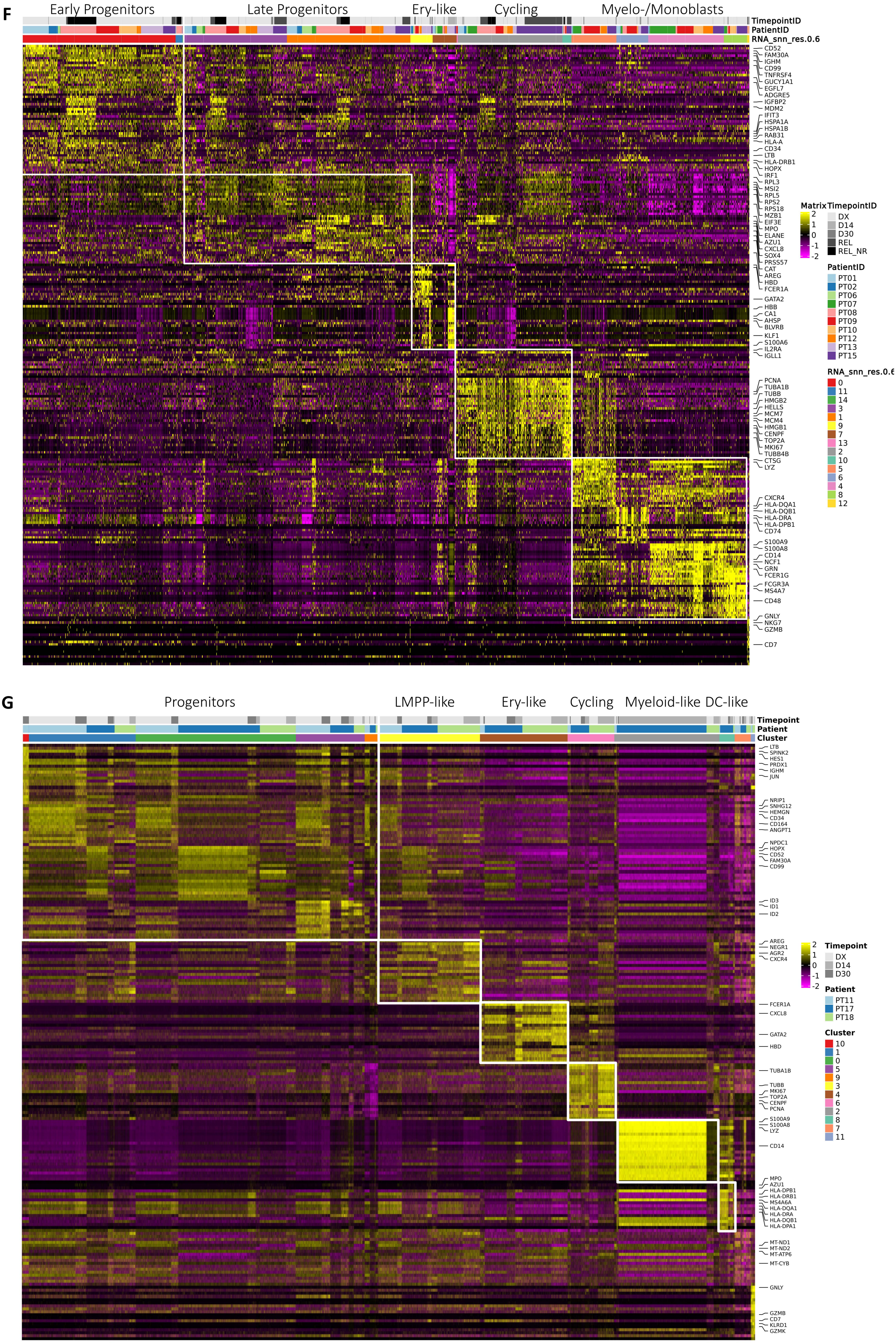

F
Early Progenitors
Late Progenitors
Ery-like
Cycling
Myelo-/Monoblasts
TimepointID
PatientID
RNA_snn_res.0.6
CD52
FAM30A
IGHM
CD99
TNFRSF4
GUCY1A1
EGFL7
ADGRE5
IGFBP2
MDM2
IFIT3
HSPA1A
HSPA1B
RAB31
HLA-A
CD34
LTB
HLA-DRB1
HOPX
IRF1
RPL3
MSI2
RPL5
RPS2
RPS18
MZB1
EIF3E
MPO
ELANE
AZU1
CXCL8
SOX4
PRSS57
CAT
AREG
HBD
FCER1A
GATA2
HBB
CA1
AHSP
BLVRB
KLF1
S100A6
IL2RA
IGLL1
PCNA
TUBA1B
TUBB
HMGB2
HELLS
MCM7
MCM4
HMGB1
CENPF
TOP2A
MKI67
TUBB4B
CTSG
LYZ
CXCR4
HLA-DQA1
HLA-DQB1
HLA-DRA
HLA-DPB1
CD74
S100A9
S100A8
CD14
NCF1
GRN
FCER1G
FCGR3A
MS4A7
CD48
GNLY
NKG7
GZMB
CD7
Matrix
2
1
0
-1
-2
TimepointID
DX
D14
D30
REL
REL_NR
PatientID
PT01
PT02
PT06
PT07
PT08
PT09
PT10
PT12
PT13
PT15
RNA_snn_res.0.6
0
11
14
3
1
9
7
13
2
10
5
6
4
8
12
G
Progenitors
LMPP-like
Ery-like
Cycling
Myeloid-like
DC-like
Timepoint
Patient
Cluster
LTB
SPINK2
HES1
PRDX1
IGHM
JUN
NRIP1
SNHG12
HEMGN
CD34
CD164
ANGPT1
NPDC1
HOPX
CD52
FAM30A
CD99
ID3
ID1
ID2
AREG
NEGR1
AGR2
CXCR4
FCER1A
CXCL8
GATA2
HBD
TUBA1B
TUBB
MKI67
TOP2A
CENPF
PCNA
S100A9
S100A8
LYZ
CD14
MPO
AZU1
HLA-DPB1
HLA-DRB1
MS4A6A
HLA-DQA1
HLA-DRA
HLA-DQB1
HLA-DPA1
MT-ND1
MT-ND2
MT-ATP6
MT-CYB
GNLY
GZMB
CD7
KLRD1
GZMK
2
1
0
-1
-2
Timepoint
DX
D14
D30
Patient
PT11
PT17
PT18
Cluster
10
1
0
5
9
3
4
6
2
8
7
11

Supplementary Figure 2

We transduced primary AML cells from 4 patients with the miR-126 reporter and analyzed xenografts (Fig. 3A). The miR-126 reporter identified intra-tumor heterogeneity (ITH), with the GFP^{low}/miR-126^{high} cell subpopulation enriched in CD34^{+}38^{-} cells (Supplementary Fig. 3C) and confirmed to express higher miR-126 RNA than its GFP^{high} counterpart (Supplementary Fig. 3D). miR-126 intra-tumor heterogeneity (ITH) was maintained over serial xenotransplantation passages and in paired diagnosis/relapse samples from PT03 (Supplementary Fig. 2A). Taking advantage of semi-randomly distributed lentiviral integration sites, we confirmed that sorted GFP^{high}/miR-126^{low} and GFP^{low}/miR-126^{high} subpopulations were clonally related, with 88% of genomic integration sites retrieved from the rare GFP^{low}/miR-126^{high} population also found in GFP^{high}/miR-126^{low} blasts. Moreover, a higher proportion of integration sites found in the GFP^{low}/miR-126^{high} population was propagated in serial transplantation assays compared to integration sites found in GFP^{high}/miR-126^{low} blasts only (Supplementary Fig. 2B,C).

A Representative FACS plots exemplifying miR-126 sensor-specific GFP downregulation and the relative enrichment in immature CD34^{+} blasts within GFP^{low}/miR-126^{high} (green histogram) vs GFP^{high}/miR-126^{low} (grey histogram) subsets across multiple patients (PT01, PT02, PT03, PT04), disease status (PT03-Diagnosis, PT03-Relapse) and serial passaging in xenografts (Passage #1,#2,#3).

B Experimental design for lentiviral vector integration site analysis in xenotransplanted AML samples. BM-derived human AML cells from primary recipients were FACS-sorted to isolate transduced cells (top) or GFP$^{high/low}$ populations (bottom). Transduced cells were then transplanted in secondary recipients. Lentiviral integrations sites (IS) were retrieved from gDNA of engrafting human cells and compared amongst groups.

C Top: Venn diagram illustrating IS sharing among primary GFP^{low}/miR-126^{high} and GFP^{high}/miR-126^{low} subsets and secondary recipient engrafting cells. Bottom: Plot reporting the percentage of shared IS.

Supplementary Figure 2:

A

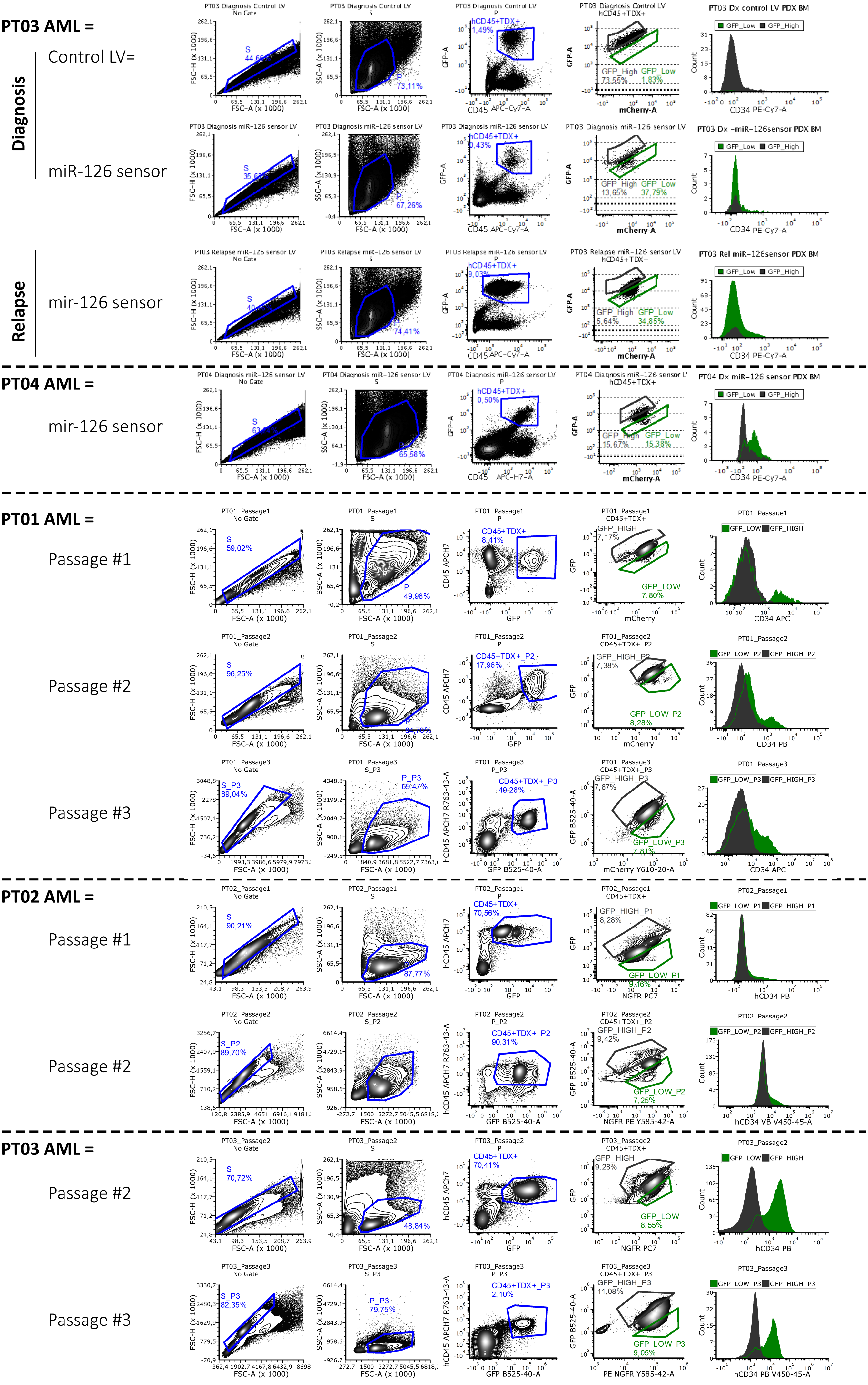

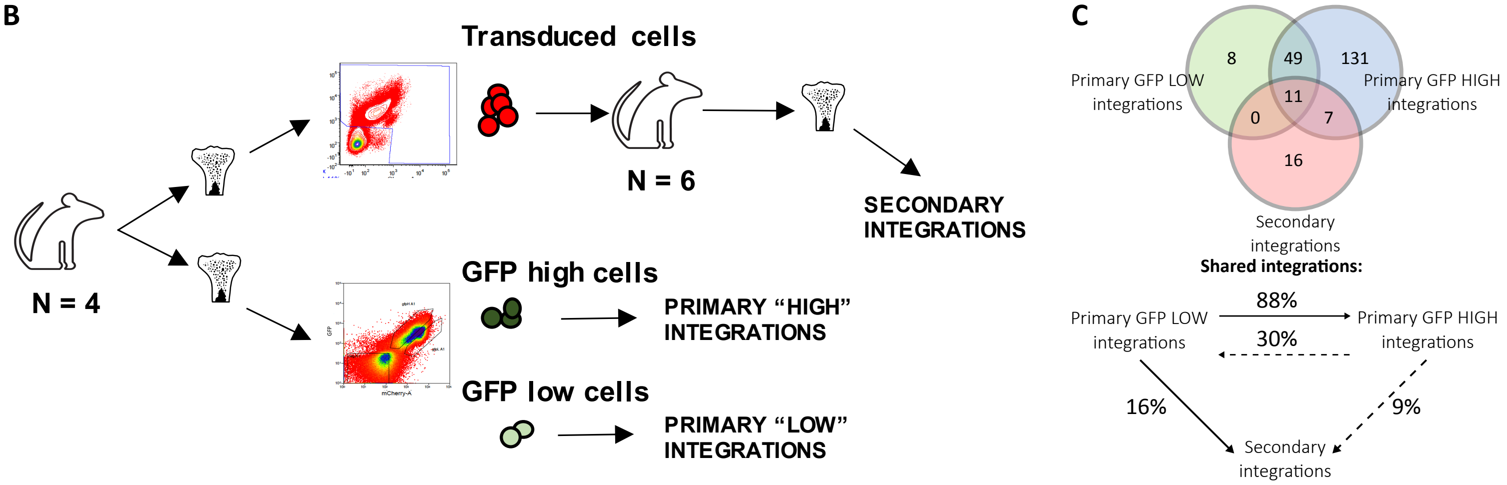

B
Transduced cells
N = 6
SECONDARY INTEGRATIONS
N = 4
GFP high cells
PRIMARY "HIGH" INTEGRATIONS
GFP low cells
PRIMARY "LOW" INTEGRATIONS
mCherry-A
GFP
C
Primary GFP LOW integrations
Primary GFP HIGH integrations
Secondary integrations
8
49
131
11
0
7
16
Shared integrations:
Primary GFP LOW integrations
88%
30%
Primary GFP HIGH integrations
16%
9%
Secondary integrations

Supplementary Figure 3

A Left**:** Table summarizing the fraction of engrafting animals per cell dose for GFP^{low}/miR-126^{high} or GFP^{high}/miR-126^{low} fractions from PT01, PT02, PT03 and PT16 xenografted AML. L.I.C., estimated leukemia initiating cell frequency. Within each disease, the fold L.I.C. enrichment in GFP^{low} over GFP^{high} subpopulations and p-value is reported. p-values were adjusted with Holm's correction to account for multiple testing. The lower limit of engrafting cell dose was not reached for PT02 at 500 cells/mouse (L.I.C. of 1/1). Engraftment was defined as >0.1% hCD45^{+} blasts in mouse BM at 16 +/- 2 weeks from transplant. n.a., not analyzable. Right**:** LDA transplantation dose response plots with estimated mean L.I.C. frequency (full line) and 95% Wald confidence intervals (dashed lines) for tested samples reported in **A**. Triangles indicate assays (transplant doses) with 100% success. For PT02, top 2 fully engrafting doses are not shown for plotting purposes.

B Flow cytometry evaluation of the percent of G0 (left), G1 (center), and G2M/S (right) blasts within sorted GFP^{low}/miR-126^{high} and GFP^{high}/miR-126^{low} subpopulations from BM aspirates of mice engrafted with PT01,PT03, and PT16 AML prior to in-vivo chemotherapy treatment. Full lines connect paired measurements from the same mice. Bottom row: Ki67 /Hoechst flow cytometry plots for discrimination of G0, G1, G2M/S cell cycle phases in GFP^{high}/miR-126^{low} and GFP^{low}/miR-126^{high} AML subpopulations recovered from exemplary PDX. n=17 PDX from 3 patients over 3 independent experiments. Data are presented as mean +/- SD.

C Relative abundance of immature CD34^{+}38^{-} blasts within GFP^{low}/miR-126^{high} or GFP^{high}/miR-126^{low} subsets revealed by miR-126 reporter in *NPM1*^{mut} AML xenografts. n=14 PDX from 4 patients over 4 independent experiments. Data are presented as standard Tukey boxplots: center: median, box: interquartile range (IQR), whiskers: IQR*1.5.

D miR-126 expression measured by digital droplet PCR (ddPCR) in sorted GFP$^{high/low}$ populations from AML xenografts. Sample pairs sorted from the same mouse are connected by full lines. miR-126 levels are expressed as normalized copies/ng input cDNA. n=27 PDX from 4 patients over 4 independent experiments. Boxplot representation same as **C**.

E AML engraftment in pre-treatment BM aspirates of xenotransplanted NSGW41 mice. Percentage of transduced hCD45^{+} cells on total physical gate cells by flow cytometry is reported. For PT01 and PT03 two independent experimental replicates are represented by different shapes (circles: replicate A, squares: replicate B). n=77 PDX from 5 patients over 7 experiments. Boxplot representation same as **C**.

F Overall transgene ratio (TGR) (mean fluorescence intensity (MFI) ratio of GFP over mCherry or NGFR) of human, transduced BM AML cells at day 8 from start of treatment. n=78 PDX from 5 patients over 7 independent experiments. Boxplot representation same as **C**.

G TransGene ratio (GFP MFI/mCherry MFI or GFP MFI/NGFR MFI) density histogram of BM AML at single-cell resolution for each xenograft at day 8 from start of treatment. n=56 PDX from 5 patients over 5 independent experiments.

H Flow cytometry evaluation of the percent of G0 BM blasts from PT01 and PT03 PDX at baseline (B/L) or after in-vivo chemotherapy (Treat). Full lines connect paired measurements from the same mice. Right: Ki67/Hoechst flow cytometry plots for discrimination of G0, G1, G2M/S cell cycle phases prior to and after in vivo chemotherapy in two exemplary PDX. n=24 PDX from 2 patients over 2 independent experiments. Data are presented as mean +/- SD.

For panels **B**, **C**, **D** and **H** Comparisons between paired groups were performed with the paired two-sided Wilcoxon test. Since for n=5, the minimum achievable two-sided p-value of the test is 0.0625, another non-parametric test based on bootstrap sampling was employed for conditions with such sample size and such p-values are italicized. n.s.: p-value >0.05.
For panels **E** and **F**: comparisons between groups were performed by using linear mixed-effects (LME) models to account for mice with the same donor and for experimental replicates. p-values of post-hoc comparisons per patient were adjusted with Holm's correction in order to account for multiple testing. ns: not significant.
Source data are provided as a Source Data file.

Suplementary Figure 3:

A

			PT01		PT02		PT03		PT16	
GFP population:			Low	High	Low	High	Low	High	Low	High
cell dose	4-5	* 10^5	3/3	2/3	3/3	3/3				
	1-2		4/4	0/4	3/3	4/4	3/3	3/3	5/5	6/6
	5	* 10^4	3/4	1/4			2/2	3/3	5/5	4/4
	2				5/5	5/5				
	1				1/1	1/2			4/5	2/6
	5	* 10^3	2/5	0/5	6/6	3/5	4/5	0/4		
	1				5/5	1/5				
	5	* 10^2			3/3	0/5	0/5	0/5		
L.I.C. Frequency			1/23518	1/481608	1/1	1/6823	1/3849	1/24469	1/6190	1/17621
Fold enrichment			20.5		n.a.		6.4		2.85	
p-value:			8.75 e-06		2.60 e-08		0.036		0.1526	
Adj. p-value:			2.63 e-05		1.04 e-07		0.072		0.1526	

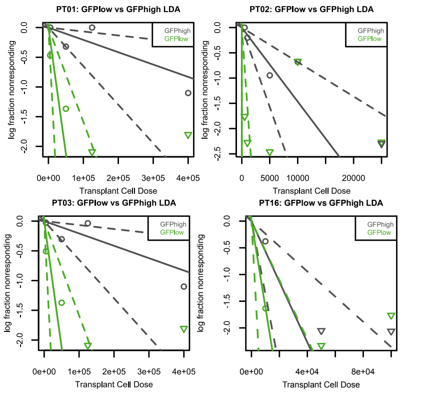

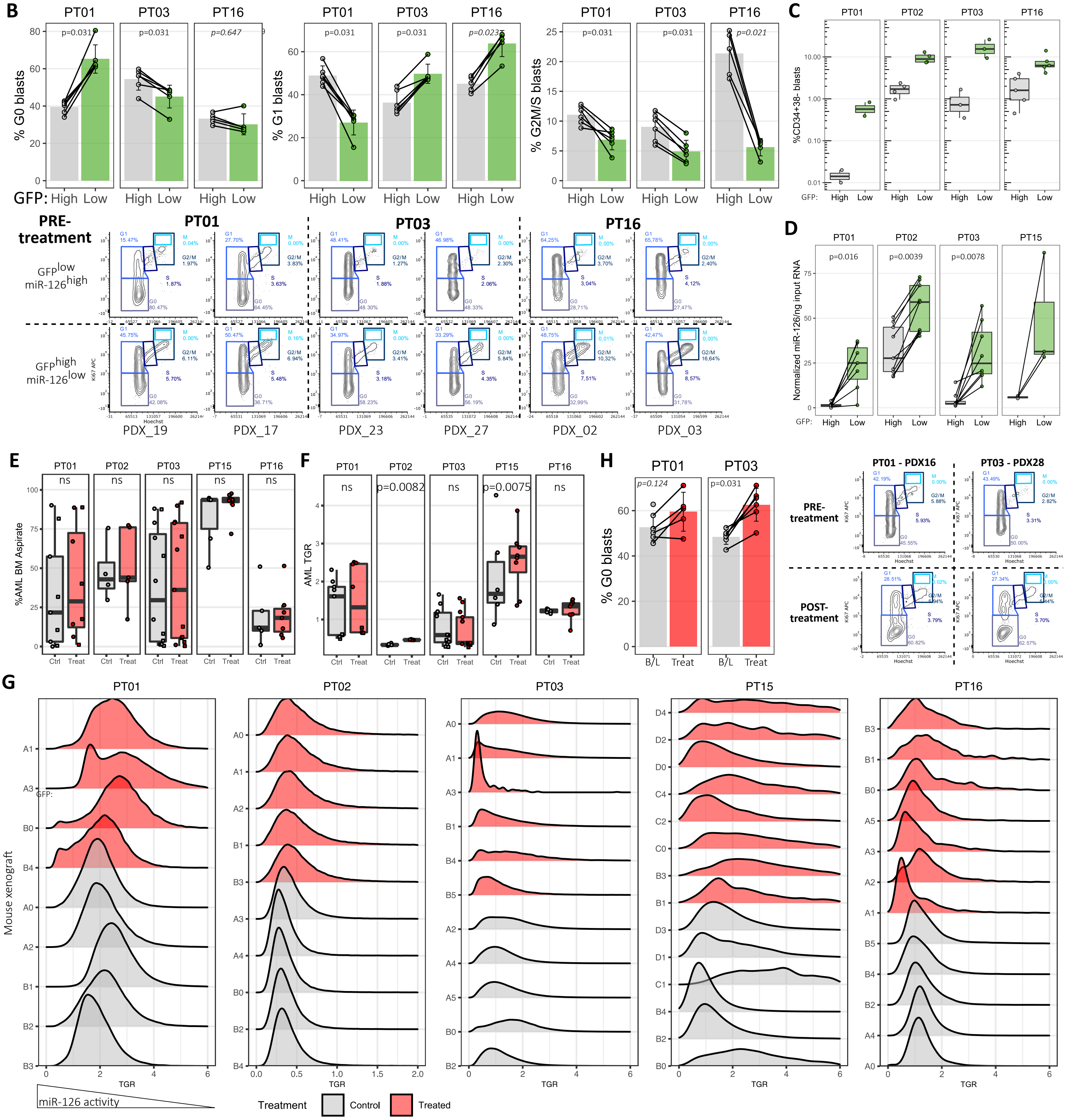

Supplementary Figure 4

A Exemplary flow cytometry gating and sorting strategy for enrichment of AML cells from BM of xenografts at day 8 from start of treatment. Gating strategy: Singlets/Physical/human $CD45^+$ & murine $CD45^-$ cells were sorted for single-cell RNA sequencing and limiting dilution transplantation of chemotherapy-treated and control AML; miR-126 reporter transduced blasts (Transduced+) were brought forward and separated in top 15-20% GFP^{high} & GFP^{low} subpopulations for bulk RNA sequencing of both subpopulations as well as single-cell RNA sequencing of GFP^{low} blasts.

B Expression of *ADGRG1* gene (normalized counts) for sequenced GFP^{low}/$miR\text{-}126^{high}$ (green, L) and GFP^{high}/$miR\text{-}126^{low}$ (grey, H) samples from control (C) or chemotherapy treated (T) AML patient xenograft. n=31 PDX from 4 patients over 4 independent experiments. Data are presented as standard Tukey boxplots: center line: median, box: interquartile range, whiskers: IQR*1.5.

C Expression of LSC17 score for sequenced GFP^{low}/$miR\text{-}126^{high}$ (green, L) and GFP^{high}/$miR\text{-}126^{low}$ (grey, H) samples from control (C) or chemotherapy treated (T) AML patient xenograft. LSC17 score was calculated for each cell by multiplying each gene's rlog expression by the reported coefficient[2]. n=31 PDX from 4 patients over 4 independent experiments. Boxplot representation same as **B**.

D Heatmap of lymphoid-related genes within lymphoid Gene Ontologies - Biological Process (BP) categories enriched in $miR\text{-}126^{high}$ samples from the over-representation analysis (ORA) on DEGs from the GFP^{low}/$miR\text{-}126^{high}$ vs GFP^{high}/$miR\text{-}126^{low}$ bulk RNA sequencing comparison. Rows are genes, while columns represent single sorted samples. Treatment group, patient ID, and GFP sorting gate are annotated on the top of the heatmap.

E Heatmap of agglomerated z-scores for enriched Hallmark gene sets (MSigDB H group) (rows) from the chemotherapy-treated vs. control comparison in GFP^{high}/$miR\text{-}126^{low}$ only blasts. Each column represents a single sorted sample. Treatment group, AML patient ID and GFP population (GFP^{high}) are annotated on top.

Source data are provided as Source Data file.

Supplementary Figure 4:

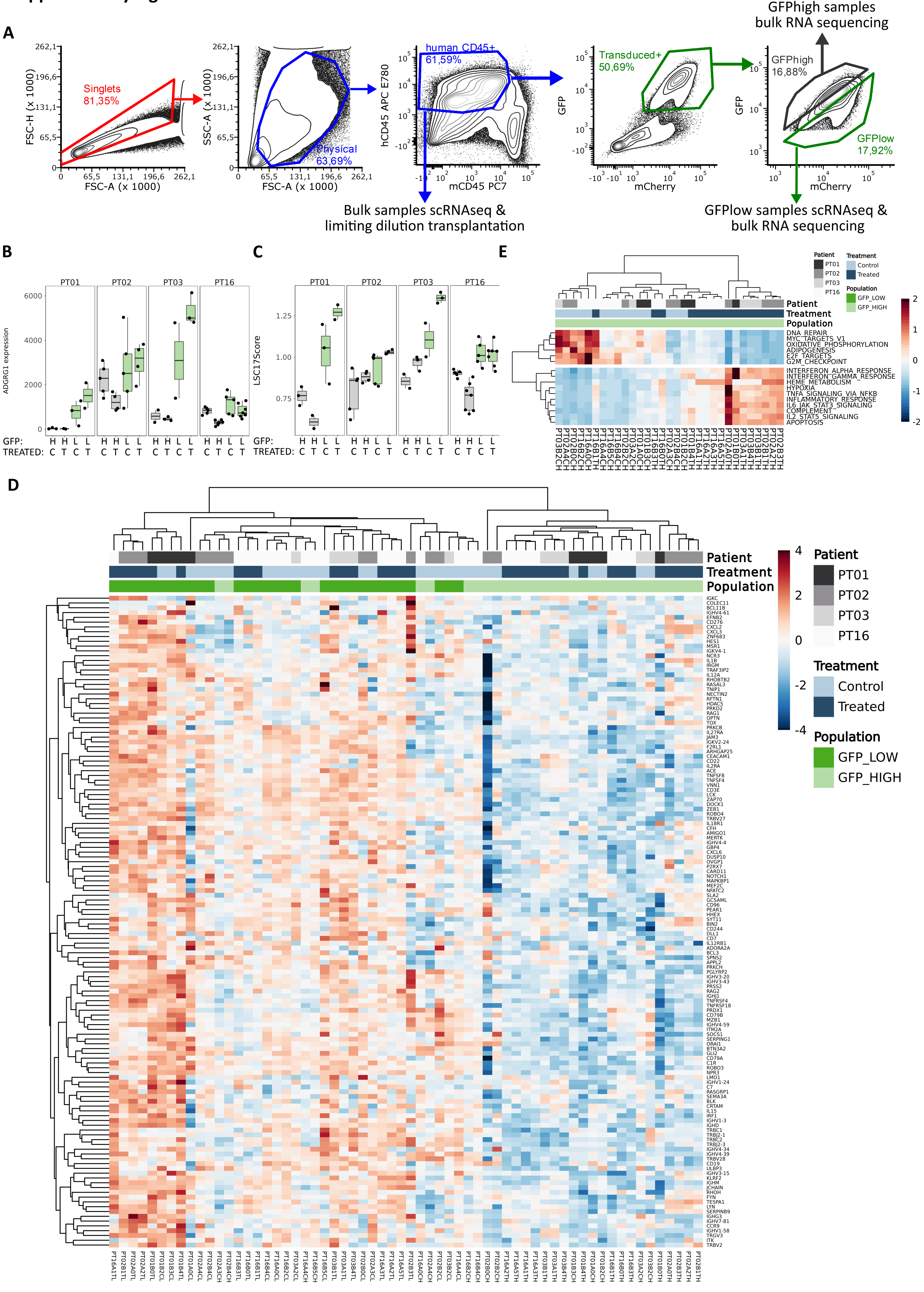

Supplementary Figure 5

A Left: UMAP plot of the total merged single-cell RNA sequencing (scRNA-seq) xenograft AML dataset prior to the harmonization process[1]. Right: UMAP plot of the xenograft AML scRNA-seq dataset post harmonization process for correction of patient-derived variability. Cells are colored by Patient ID.

B, C UMAP plot of the xenograft AML scRNA-seq dataset colored by reference-based cell annotations.

D UMAP plot of xenograft AML scRNA-seq cells colored by unsupervised clustering (resolution = 1.2). The dashed line highlights localization of Cluster 1 HSC-like cells at resolution = 0.6.

E TransGene Ratio of miR-126 sensor transduced AML blasts after in-vitro culture for 4 days exposed to Cytarabine (ARA, 50 ng/ml), IACS-010759 (IACS, 30 μM), both (ARA_IACS), or mock treatment (MOCK). n=4 technical replicates per condition.

F In vitro growth curves of miR-126 sensor transduced AML blasts exposed to Cytarabine (ARA, 50 ng/ml), IACS-010759 (IACS, 30 μM), both (ARA_IACS), or mock treatment (MOCK). n=4 technical replicates per condition.

G Percent of CD34+ blasts after in-vitro culture for 4 days exposed to Cytarabine (ARA, 50 ng/ml), IACS-010759 (IACS, 30 μM), both (ARA_IACS), or mock treatment (MOCK). n=4 technical replicates per condition. Data are presented as standard Tukey boxplots: center line: median, box: interquartile range, whiskers: IQR*1.5.

Source data are provided as Source Data file.

Supplementary Figure 5:

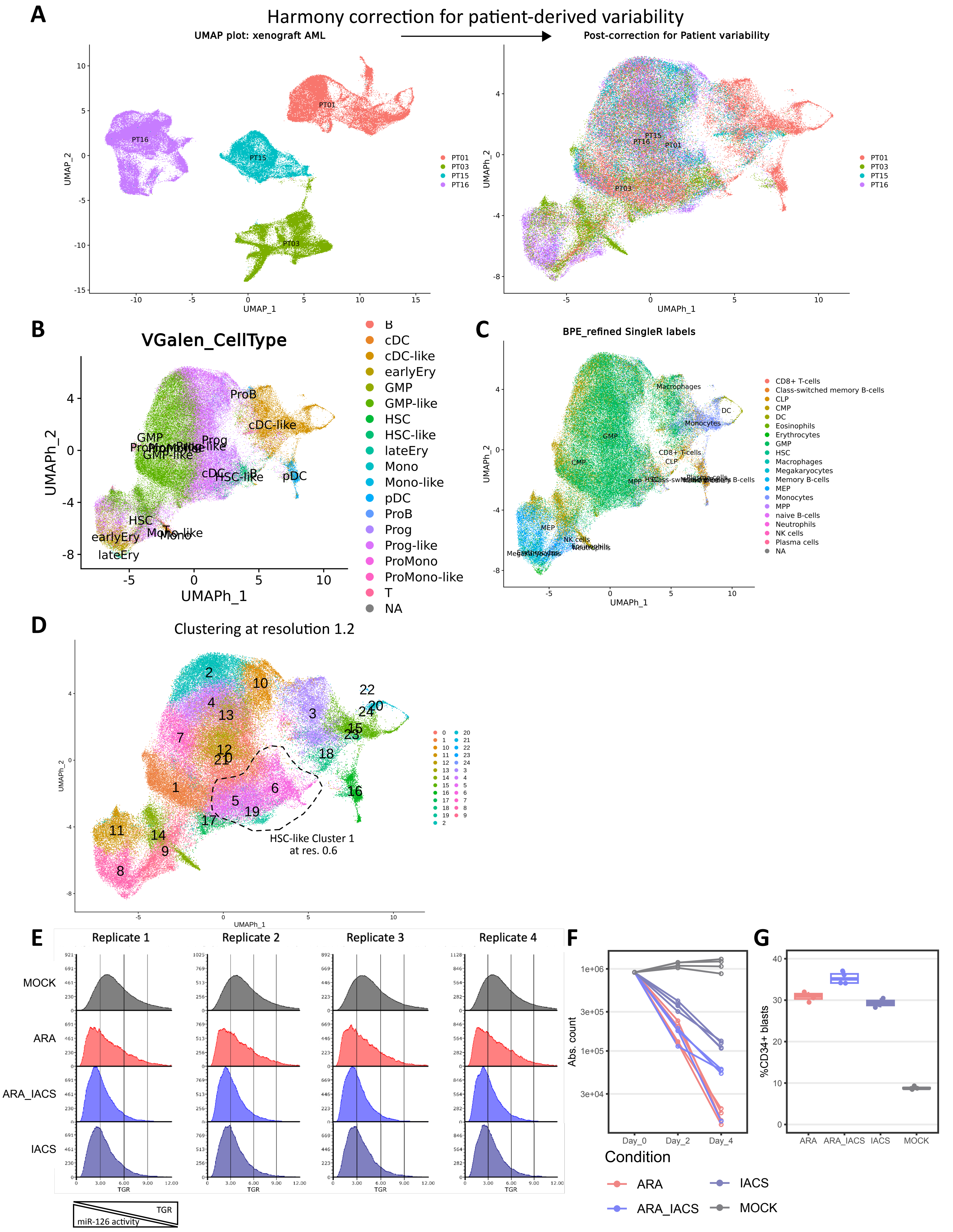

Supplementary Figure 6

A Correlation between module scores for LSC- and lymphoid-gene subsets. Cells are colored by patient outcomes (dark red for primary refractory, orange for early relapse after initial CR, green for persistent CR patients). Spearman rho correlation coefficient and p value are reported. Left: *NPM1*^{mut} AML dataset. Right: del(7) AML dataset.

B Mapping of the expression of the 126Low module score on UMAP plots of *NPM1*^{mut} (left) or del(7) (right) AML cells.

C Mapping of the LSC17 gene signature on UMAP plots of NPM1^{mut} (left) or del(7) (right) AML cells. LSC17 score was calculated for each cell by multiplying each gene's scaled expression by the reported coefficient[2].

D Kaplan-Meier plot with log rank test p-value for overall survival of AML patients from gene expression data of the microarray GSE1247 & GSE37642 (top), TCGA-AML (middle), and OSHU (bottom*)* cohorts. Log rank test p-value and Cox's proportional hazards model with lasso penalty hazard ratios (HR) with 95% confidence interval are reported. Risk groups are identified as follows: black line, when the value of the linear combination of selected genes within the 126High restricted gene list is less or equal to the median, red line when it is above the median.

E Single-cell expression heatmap of the top 10 marker genes of each LSC subcluster from the *NPM1*^{mut} AML dataset. Each column represents a single cell transcriptome and is annotated on top for LSC subcluster, patient and sampling timepoint variables.

Supplementary Figure 6:

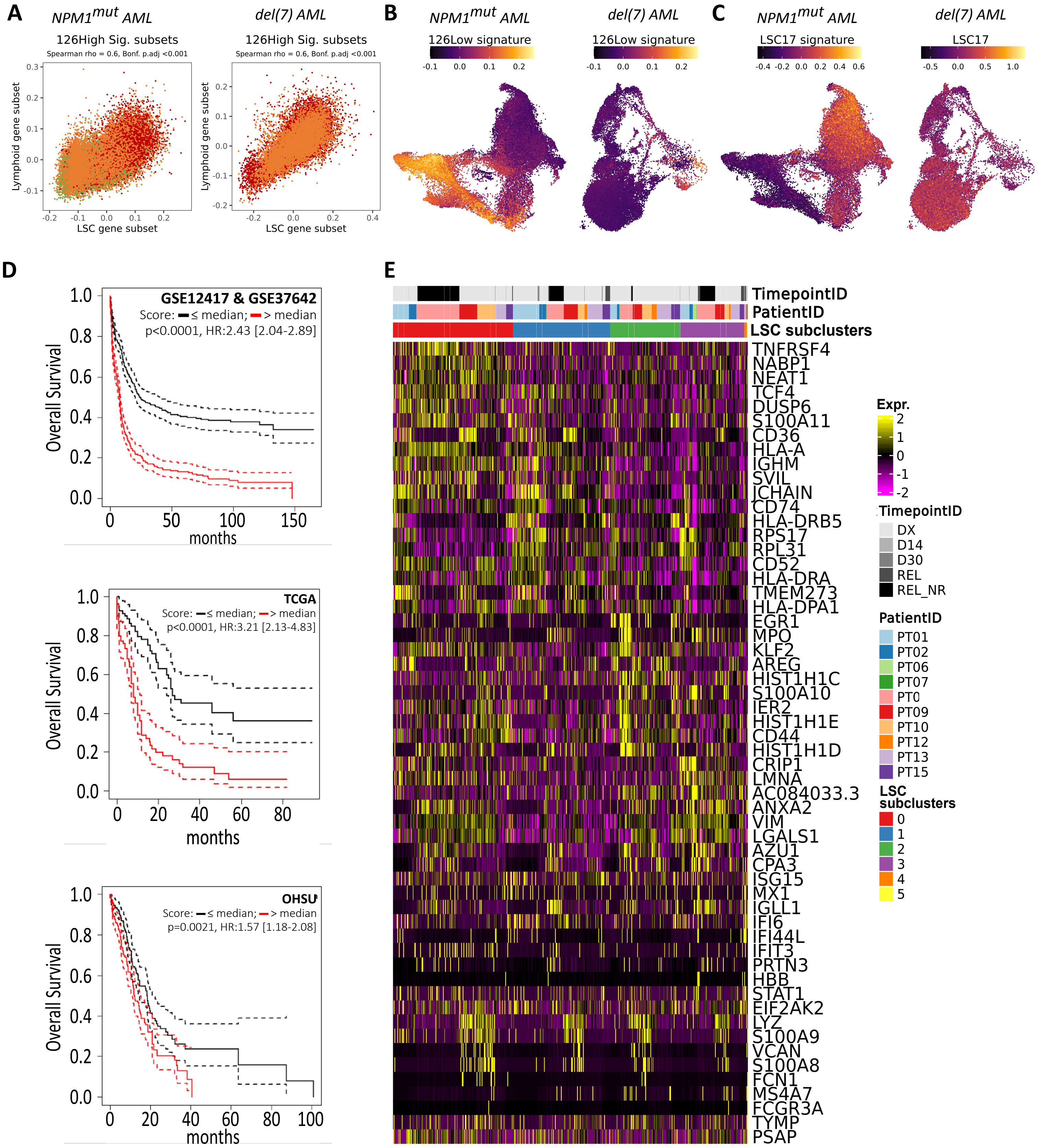

Supplementary Figure 7

A Top to bottom: PT01, PT02, PT06, PT07. Left: UMAP plot of each single patient's BMMCs (PBMCs for diagnosis of PT06) from all sampled timepoints in scRNAseq passing quality control filtering and merged together. Cells are colored by cluster from unsupervised clustering at resolution 3.0. Middle: Heatmap reporting percent composition of each NPM1-MF category (*NPM1*^{mut} (MUT); wild type (WT), Not Detected (ND); No Call) in each cluster identified at resolution 3.0. Left "AML" column denotes whether cells from that cluster (excluding those defined as WT) were considered as AML for further analysis based on NPM1-MF criteria (See methods: *NPM1* Mutation Finder). Right: Heatmap reporting percent composition of SingleR-based cell classification categories based on the BluePrint Encode main reference in each cluster identified at resolution 3.0.

B Same as **A** for patients: PT08, PT09, PT10, PT12 (ordered top to bottom).

C Same as **A** for patients: PT13, PT15, PT19, PT20 (ordered top to bottom)

D Top to bottom: PT11, PT17, PT18. Distribution of Chromosome 7 module score values computed on the expression of genes located on Chromosome 7 in cells classified as AML (red), or non AML (blue) by k-means clustering (k = 2).

Supplementary Figure 7:

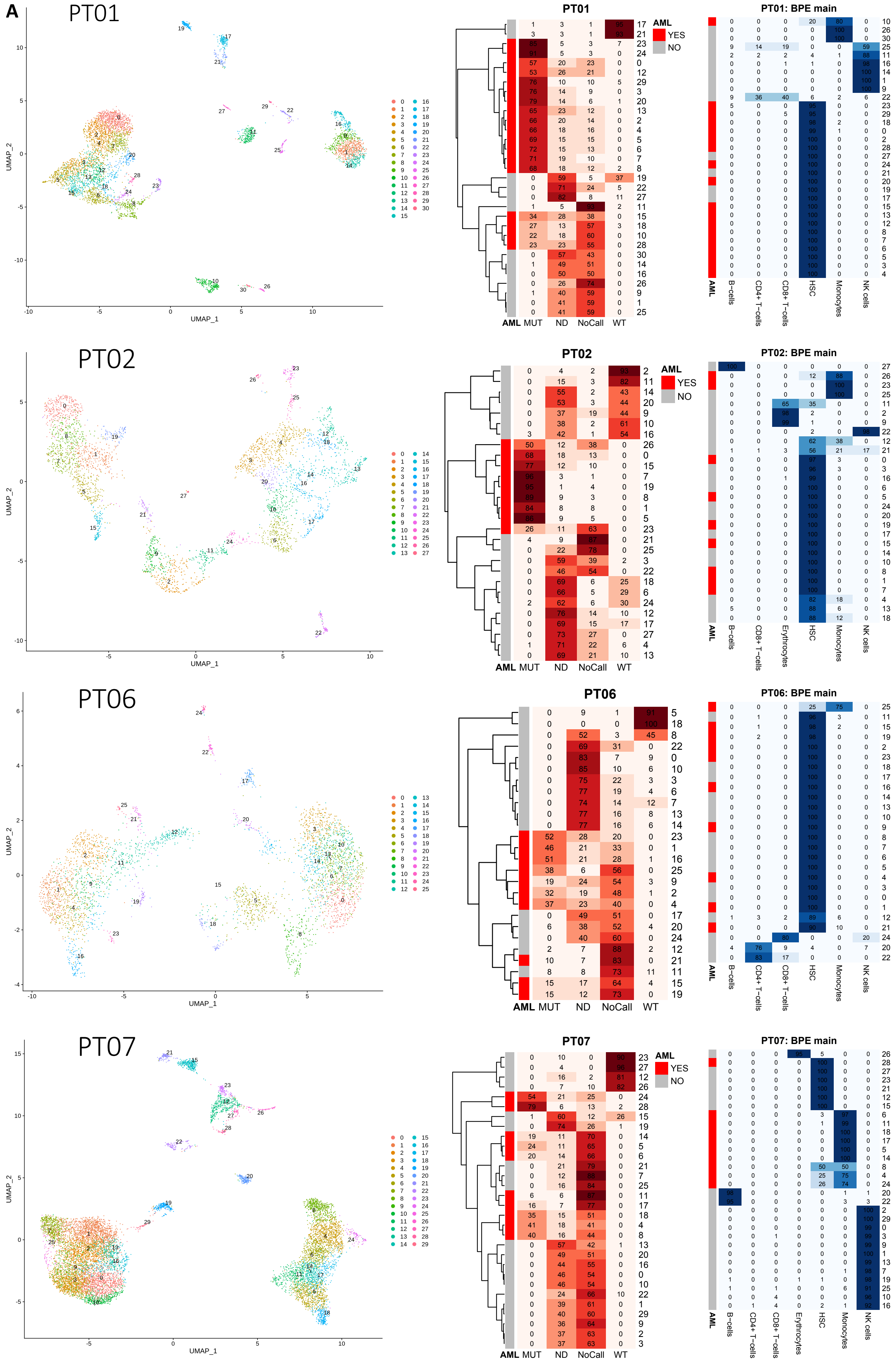

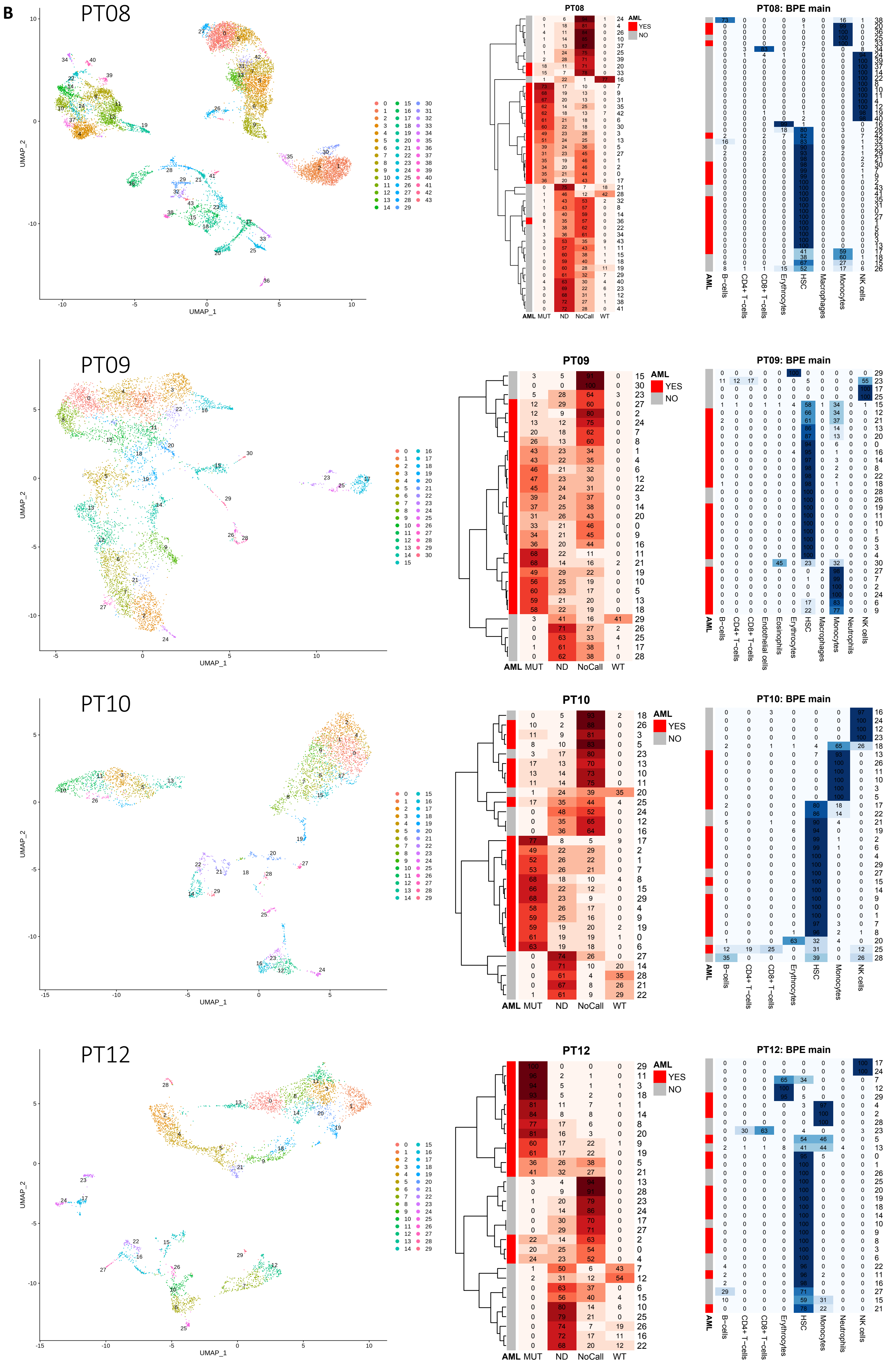
B
PT08
PT08: BPE main
PT09
PT09: BPE main
PT10
PT10: BPE main
PT12
PT12: BPE main
UMAP_1
UMAP_2
AML
YES
NO
AML MUT ND NoCall WT
B-cells
CD4+ T-cells
CD8+ T-cells
Endothelial cells
Eosinophils
Erythrocytes
HSC
Macrophages
Monocytes
Neutrophils
NK cells

C

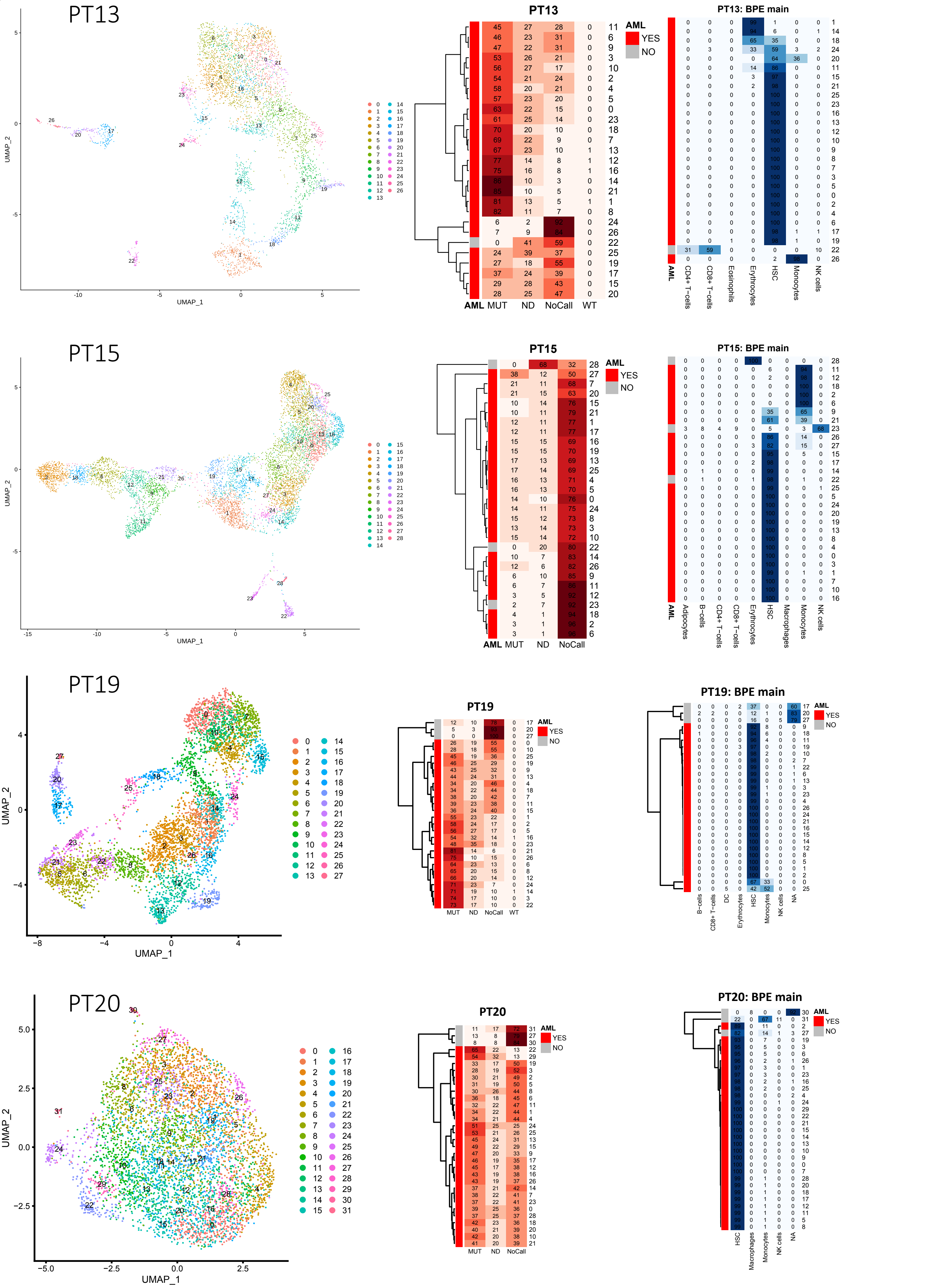

PT13
UMAP_1
UMAP_2
PT13
AML
YES
NO
MUT
ND
NoCall
WT
PT13: BPE main
CD4+ T-cells
CD8+ T-cells
Eosinophils
Erythrocytes
HSC
Monocytes
NK cells
PT15
PT15: BPE main
Adipocytes
B-cells
Macrophages
PT19
PT19: BPE main
DC
NA
PT20
PT20: BPE main

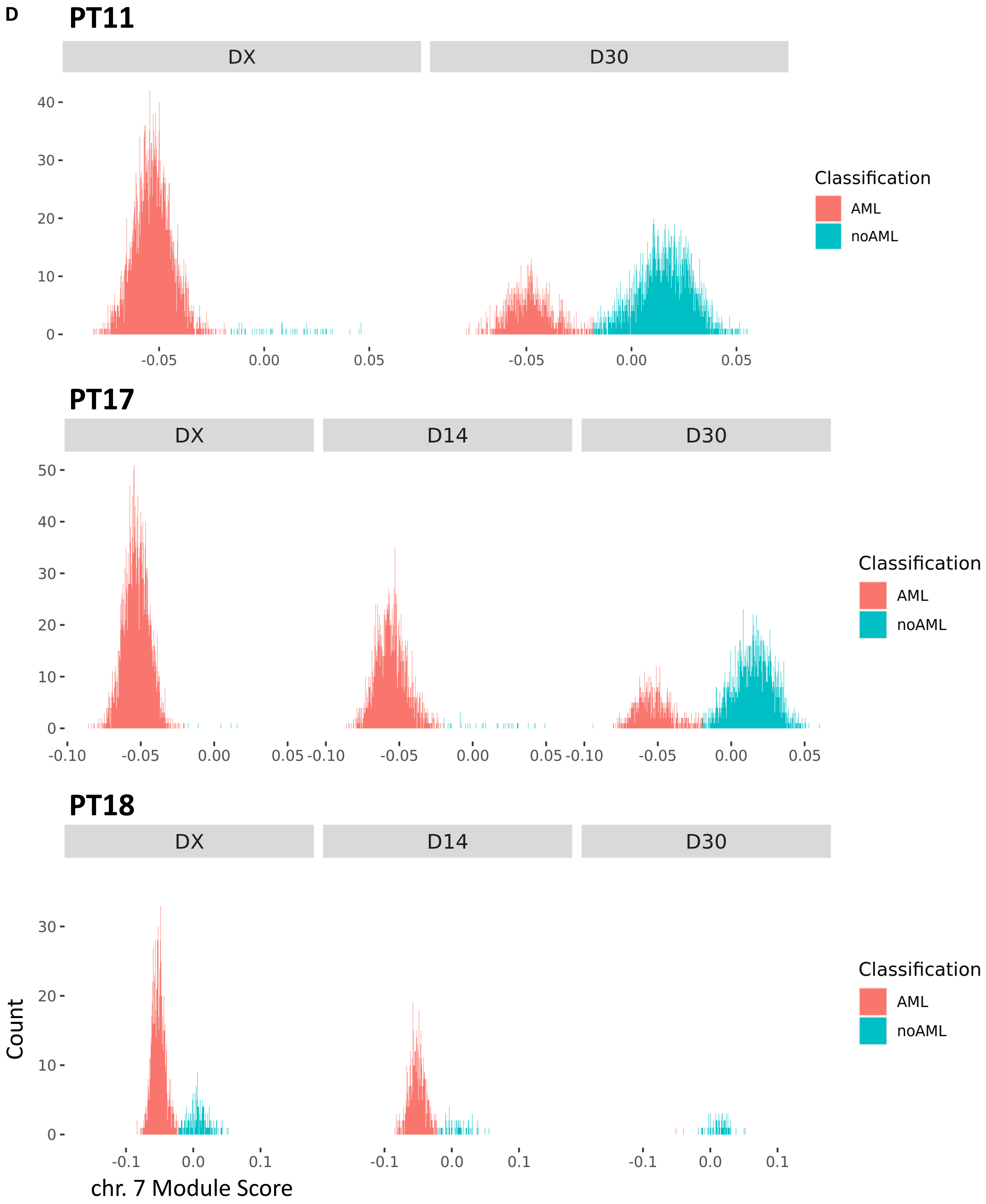
D
PT11
DX
D30
40
30
20
10
0
-0.05
0.00
0.05
Classification
AML
noAML
PT17
DX
D14
D30
50
-0.10
PT18
0.1
0.0
-0.1
Count
chr. 7 Module Score

Supplementary Figure 8

A Heatmap of k-scores for semantic similarities across Gene Ontology Biological Process enriched categories in miR-126^{high} blasts in bulk RNA sequencing comparing miR-126^{high} vs miR-126^{low} subpopulations.

B Heatmap of k-scores for semantic similarities across Gene Ontology Biological Process enriched categories in miR-126^{low} blasts after a pruning step to reduce redundant labels in bulk RNA sequencing comparing miR-126^{high} vs miR-126^{low} subpopulations.

REFERENCES:

1. Korsunsky, I. *et al.* Fast, sensitive and accurate integration of single-cell data with Harmony. *Nat. Methods* **16**, 1289–1296 (2019).
2. Ng, S. W. K. *et al.* A 17-gene stemness score for rapid determination of risk in acute leukaemia. *Nature* **540**, 433–437 (2016).

Supplementary Figure 8:

A

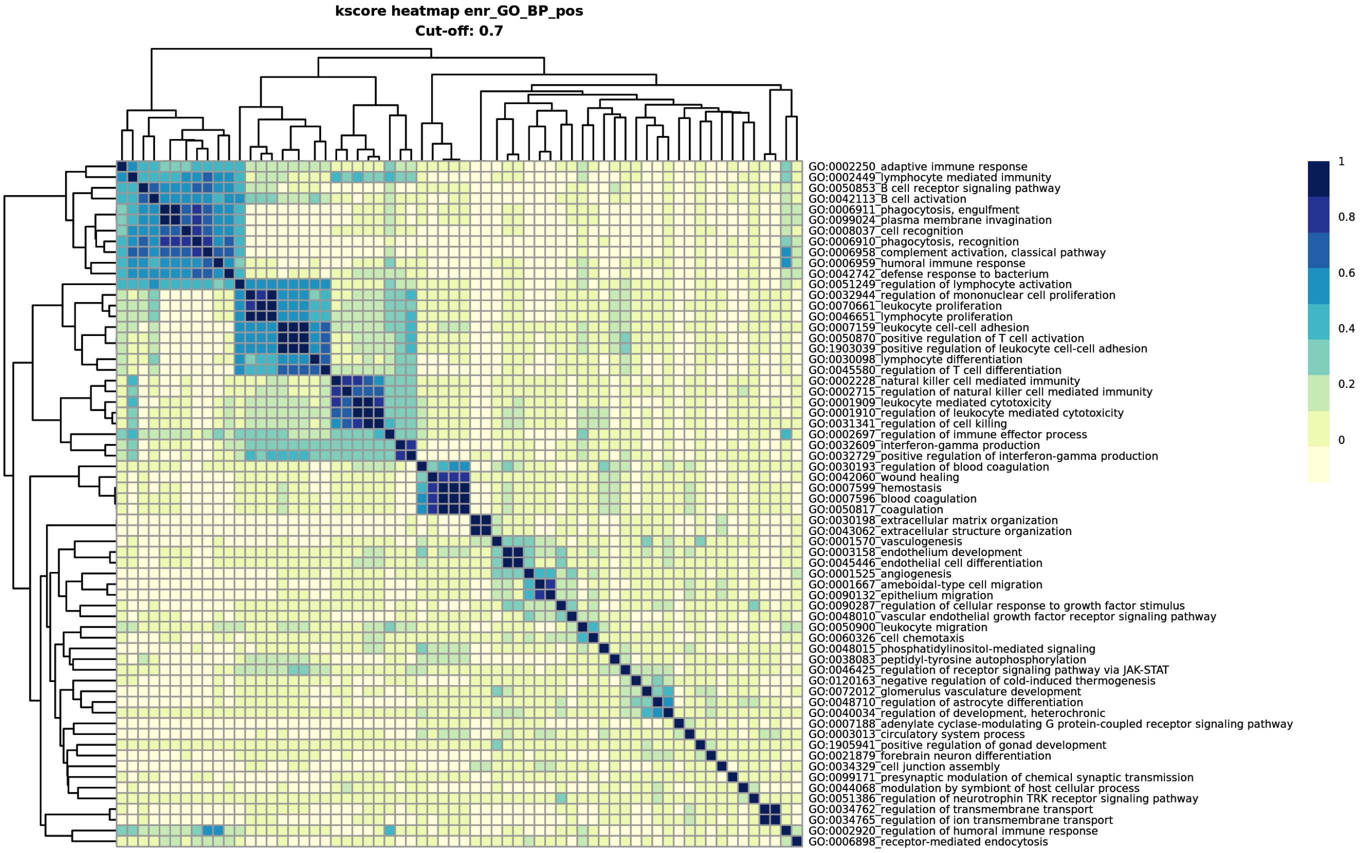

B

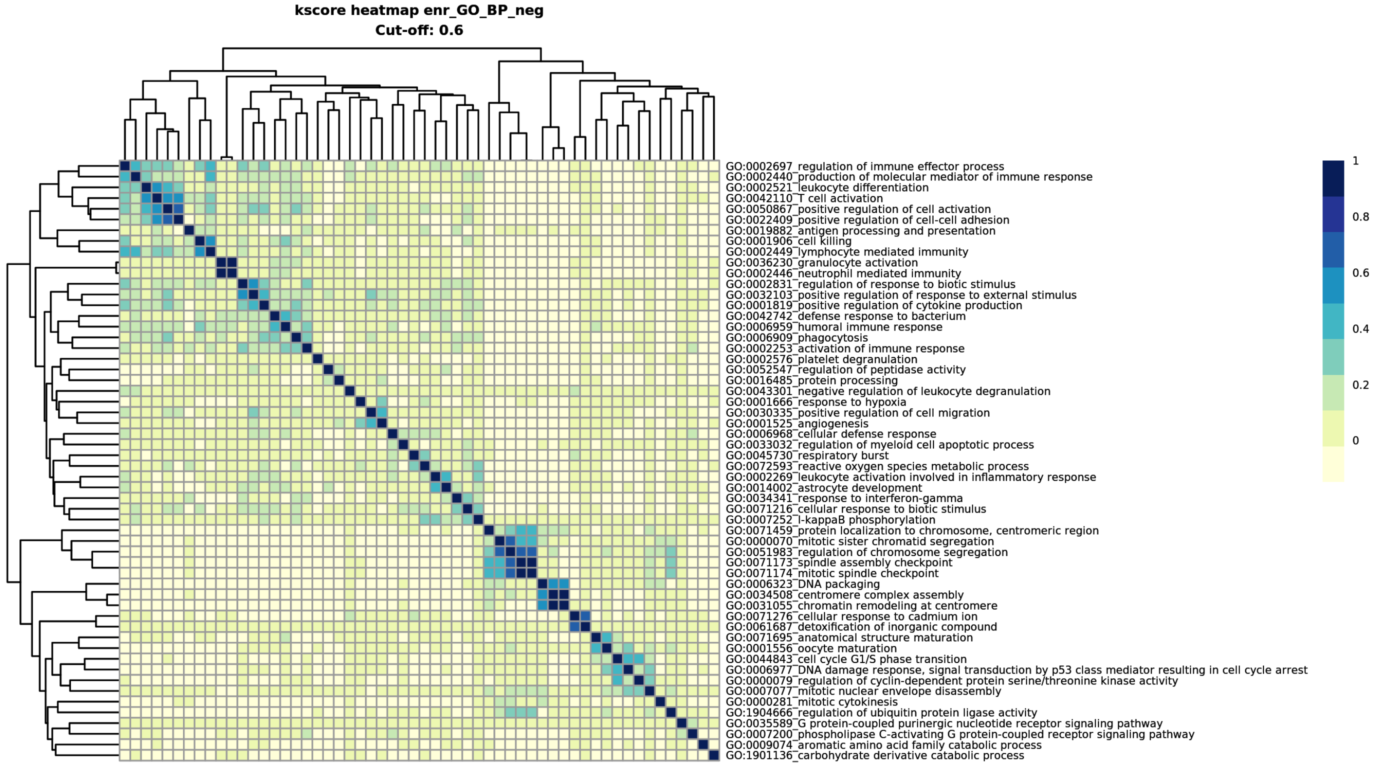

Supplement: Supplementary file 1 — Supplementary Information [file 41467_2023_36969_MOESM1_ESM.pdf]
