# Supplementary material for: Longitudinal single-cell profiling of chemotherapy response in acute myeloid leukemia
Source: Nat Commun. 2023 Mar 8;14:1285. doi: 10.1038/s41467-023-36969-0 (PMC9995364; doi:10.1038/s41467-023-36969-0)
Supplement: Supplementary file 3 — Reporting Summary [file 41467_2023_36969_MOESM3_ESM.pdf]

nature portfolio

Corresponding author(s): Bernhard Gentner

Last updated by author(s): Feb 3, 2023

Reporting Summary

Nature Portfolio wishes to improve the reproducibility of the work that we publish. This form provides structure for consistency and transparency in reporting. For further information on Nature Portfolio policies, see our Editorial Policies and the Editorial Policy Checklist.

Statistics

For all statistical analyses, confirm that the following items are present in the figure legend, table legend, main text, or Methods section.

n/a	Confirmed	
☐	☒	The exact sample size (*n*) for each experimental group/condition, given as a discrete number and unit of measurement
☐	☒	A statement on whether measurements were taken from distinct samples or whether the same sample was measured repeatedly
☐	☒	The statistical test(s) used AND whether they are one- or two-sided<br>*Only common tests should be described solely by name; describe more complex techniques in the Methods section.*
☐	☒	A description of all covariates tested
☐	☒	A description of any assumptions or corrections, such as tests of normality and adjustment for multiple comparisons
☐	☒	A full description of the statistical parameters including central tendency (e.g. means) or other basic estimates (e.g. regression coefficient) AND variation (e.g. standard deviation) or associated estimates of uncertainty (e.g. confidence intervals)
☐	☒	For null hypothesis testing, the test statistic (e.g. *F*, *t*, *r*) with confidence intervals, effect sizes, degrees of freedom and *P* value noted<br>*Give P values as exact values whenever suitable.*
☒	☐	For Bayesian analysis, information on the choice of priors and Markov chain Monte Carlo settings
☐	☒	For hierarchical and complex designs, identification of the appropriate level for tests and full reporting of outcomes
☐	☒	Estimates of effect sizes (e.g. Cohen's *d*, Pearson's *r*), indicating how they were calculated

Our web collection on statistics for biologists contains articles on many of the points above.

Software and code

Policy information about availability of computer code

Data collection	Digital droplet PCR was performed using the QX200 Droplet Digital PCR System (Bio-Rad). Bulk RNA sequencing libraries were produced with SMART-Seq v4 Ultra Low Input RNA Kit (Takara Bio USA, Mountain View, CA, USA), as per manufacturer’s instructions. Single cell RNA sequencing was performed with 10x genomics Gene expression v2 or v3 kits. Next Generation Sequencing was performed on Illumina platforms NextSeq or NovaSeq S1 or S2.<br>Flow cytometry data was collected on the BD FACSDiva software v.8.0.2 for FACSCanto II, LSRFortessa II FACSymphony A5. Data was collected on the CytExpert software v2.4.0.28 (Beckman Coutier) for Cytoflex S or LX.
Data analysis	Bulk RNA sequencing data quality check was performed with fastQC, preprocessing including trimming and adapter removal using TrimGalore (v0.5.0). Trimmed reads were mapped to the GRCh38 reference genome assembly provided by the 10X reference data repository (refdata-cellranger-GRCh38-3.0.0) using STAR (v2.7.0d). Post-alignment metrics, including coverage distribution across gene length and percentage of reads mapping to exons were collected by using Qorts (v1.3.6). We assigned reads to genes by using featurecounts (v1.6.3). Data preprocessing, exploration and differential gene expression (DGE) analyses were performed with DESeq2 (v1.26.0) R packages. Over Representation Analysis (ORA) and Gene Set Enrichment Analysis (GSEA) performed in clusterProfiler v3.8.1 R package. Enriched terms semantic similarity was evaluated with GOSemSim package (v2.10.0). Single cell RNA sequenced libraries were de-multiplexed and processed by Cell Ranger Single-Cell Software Suite (version 3.1.0, 10X Genomics) using GRCh38 reference genome assembly and gene transcript file annotations provided by the 10X reference data repository (refdata-cellranger-GRCh38-3.0.0). Data integration was performed with Harmony package (v1.0). Analysis was performed with Seurat R package (version 3.2.3), SingleR package (v1.1.11), Monocle package (version 3). Cox regression with lasso was performed with glmnet package (v4.0-2), LME with nlme package (v.3.1-142), post-hoc comparisons with phia package (v0.2-1), boot strap procedure with MKinfer package (v0.6), log-rank test for survival analysis with survival package (v3.2-3).<br>Flow cytometry data was analyzed with FCS Express v6 and v7 (DeNovo Software).

Code availability: Code used for the generation of results reported in this manuscript is available at the following GitLab repository [http://www.bioinfotiget.it/gitlab/custom/mnaldini_natcomm2023].

For manuscripts utilizing custom algorithms or software that are central to the research but not yet described in published literature, software must be made available to editors and reviewers. We strongly encourage code deposition in a community repository (e.g. GitHub). See the Nature Portfolio guidelines for submitting code & software for further information.

Data

Policy information about availability of data

All manuscripts must include a data availability statement. This statement should provide the following information, where applicable:

- Accession codes, unique identifiers, or web links for publicly available datasets
- A description of any restrictions on data availability
- For clinical datasets or third party data, please ensure that the statement adheres to our policy

Data availability: RNA sequencing data generated in this study have been deposited in the GEO database under accession code GSE185993 [https://www.ncbi.nlm.nih.gov/geo/query/acc.cgi?acc=GSE185993]. Source data are provided with this paper. In addition, all processed single-cell RNA sequencing data can be accessed and queried through our online, interactive user interface [http://www.bioinfotiget.it/ mnaldini_natcomm2023/]. All data accessed from external sources and prior publications have been referenced in the text, GSE12417 [https://www.ncbi.nlm.nih.gov/geo/query/acc.cgi?acc=GSE12417], GSE37642 [https://www.ncbi.nlm.nih.gov/geo/query/acc.cgi?acc=GSE37642].

Human research participants

Policy information about studies involving human research participants and Sex and Gender in Research.

Reporting on sex and gender	No selection based on sex or gender was applied. Overall, samples from 9 female patients and 9 male patients were analyzed or employed for experiments.
Population characteristics	All AML patients selected in this study were deemed fit for intensive chemotherapy with curative intent at diagnosis. Only patients affected by NPM1 mutated AML or AML with monosomy of chromosome 7 were selected in order to distinguish AML blasts from residual normal hematopoiesis upon single cell RNA sequencing. Patient characteristics are reported in Supplementary Figure 1B.
Recruitment	Patients were recruited based on the availability of peripheral blood or bone marrow samples collected at diagnosis, early after chemotherapy (Day 14 or Day 30) and/or at relapse and based on the molecular subtype of AML with either NPM1 mutation or monosomy of chromosome 7.
Ethics oversight	The research proposal was reviewed and approved by an institutional review board (Comitato Etico Ospedale San Raffaele di Milano, protocol N°RPC, v2 22/01/2019). Informed consent to biobanking of peripheral blood and bone marrow cells had been previously signed by all patients.

Note that full information on the approval of the study protocol must also be provided in the manuscript.

Field-specific reporting

Please select the one below that is the best fit for your research. If you are not sure, read the appropriate sections before making your selection.

[x] Life sciences [ ] Behavioural & social sciences [ ] Ecological, evolutionary & environmental sciences

For a reference copy of the document with all sections, see nature.com/documents/nr-reporting-summary-flat.pdf

Life sciences study design

All studies must disclose on these points even when the disclosure is negative.

Sample size	No sample size pre-determination was used for the experiments. Sample size for each experiment was determined by the total number of available transduced or FACS-recovered cells, which is constrained by the human source of the material and limited transduction efficiency of primary AML blasts, to be split among each experimental conditions. Whenever possible we aimed to reach n = 5 per group which is considered adeguate for carrying out non parametric statistical comparisons otherwise only descriptive statistics were reported.
Data exclusions	For bulk RNA sequencing analysis, 2 samples (PT01, mouse A3 GFPlow and mouse A1, GFPlow) along with their paired GFPhigh samples (PT01, mouse A1 and A3 GFPhigh) were excluded due to abnormal post-alignment metrics and behaviour as outliers in PCA plots and TGR distribution.<br>For single-cellRNA sequencing, cells not passing pre-established quality control metrics were excluded from further analysis as described in the methods section.<br>In each LME analysis, when necessary, an appropriate transformation was applied to the response variable and, eventually, few outliers were not included in the analysis in order to meet the assumption of normality of the residuals of the model:<br>Fig. 3E: The response variable y (absolute BM AML cells) was used with the square root transformation - one outlier was removed from the analysis: Patient PT03-Replicate B-Mouse B3- group Control.

Fig 3F: The response variable y (percent CD34+38- AML blasts) was used with the cube root transformation. Two outliers were removed from the analysis: Patient PT03-Replicate A-Mouse A3-group Treated and Patient PT16-Replicate A-Mouse B1-group Treated.
Supplementary Fig. 3F: The response variable y (AML TGR) was used with the ordered quantile normalization transformation. One outlier was removed from the analysis: Patient PT15-Replicate A-Mouse C1-group Control.
No other data or samples were excluded from the analysis.

Replication

As the vast majority of the experiments were performed with primary human patient samples, most experiments could not be repeated due to limited patient material, if experiments were repeated they were reported and duly noted. This caveat was overcome by the analysis of multiple different patient samples considered as biological replicates.
Experiments shown in Figure 3E,F,G and Supplementary Figure 3E,F,G for patients PT01 and PT03 were replicated successfully as reported in the manuscript.

Randomization

The only relevant variable to be balanced across groups was baseline engraftment prior to chemotherapy treatment in PDX models. Randomization of mice in treatment and control group was performed to achieve comparable human AML engraftment prior to treatment across groups.

Blinding

Experiments were not conducted in blind-fashion, blinding was not relevant for objective measures

Reporting for specific materials, systems and methods

We require information from authors about some types of materials, experimental systems and methods used in many studies. Here, indicate whether each material, system or method listed is relevant to your study. If you are not sure if a list item applies to your research, read the appropriate section before selecting a response.

Materials & experimental systems

n/a	Involved in the study
	☒ Antibodies
	☒ Eukaryotic cell lines
☒	☐ Palaeontology and archaeology
	☒ Animals and other organisms
	☒ Clinical data
☒	☐ Dual use research of concern

Methods

n/a	Involved in the study
☒	☐ ChIP-seq
	☒ Flow cytometry
☒	☐ MRI-based neuroimaging

Antibodies

Antibodies used

Species Antigen Clone Fluorochrome Company Catalogue number Dilution
Human CD45 HI30 APCeFluor780 Invitrogen 47-0459-42 2.5:100
Human CD45 HI30 Pacific Blue BioLegend 304029 3:100
Human CD45 HI30 PerCP-Cy5.5 BioLegend 304028 2:100
Human CD33 WM53 BV421 BD 562854 5:100
Human CD33 AC104.3E3 PE-Vio770 Myltenyi 130-101-061 2:100
Human CD33 REA775 VioBlue Myltenyi 130-111-024 2:100
Human CD33 REA775 APC Myltenyi 130-111-020 2:100
Human CD34 AC136 APC Myltenyi 130-113-176 2:100
Human CD34 AC136 PE-Vio770 Myltenyi 130-113-180 2:100
Human CD34 AC136 PE Myltenyi 130-113-179 2:100
Human CD34 AC136 VioBlue Myltenyi 130-113-182 2:100
Human CD117 104D2 PE-Cy7 BioLegend 313212 5:100
Human CD117 YB5.B8 PE BD 555714 5:100
Human CD271/NGFR ME20.4 APC-Fire750 BioLegend 345116 2:100
Human CD271/NGFR REA844 APC Myltenyi 130-112-602 2:100
Human CD38 HB-7 BV510 BioLegend 356612 1:100
Human CD38 HIT2 BV605 BioLegend 303532 2:100
Human CD38 HB-7 PerCP-Cy5.5 BioLegend 356614 1:100
Human CD3 UCHT1 PE BioLegend 300431 5:100
Human CD3 SK7 FITC BioLegend 344804 2:100
Human CD19 4G7 FITC BD 345776 3:100
Human CD19 HIB19 BV421 BD 562440 2:100
Human CD235a/GLYA REA175 FITC Myltenyi 130-177-688 2:100
AnnexinV NA Pacific Blue BioLegend 640918 5:100
AnnexinV NA FITC BioLegend 640906 5:100
Mouse CD45 30-F11 APC invitrogen 17-0451-82 1:100
Mouse CD45 REA737 APC-Vio770 Myltenyi 130-110-662 2:100
Mouse CD45.1 A20 BV650 BioLegend 110735 1:100
Mouse CD45.1 A20 PE BD 553776 1:100

Mouse CD45.1 A20 FITC BD 553775 1:100
Mouse Ki-67 B56 Alexa Fluor 647 BD 558615 2:100

Validation

All antibodies used in this study are commercially available. They have been used according with manufacturer instructions provided in the data-sheets available at the manufacturer's website at the reported link below or at the dilution specified above after in-house titering

Human CD45 HI30 APCeFluor780 Invitrogen 47-0459-42 2.5:100
https://www.thermofisher.com/antibody/product/CD45-Antibody-clone-HI30-Monoclonal/47-0459-42#:~:text=47%2D0459%2D42%20was%20used%20in%20Flow%20Cytometry%20to%20reveal,to%20re%2Dengage%20viral%20defence.&text=Characterization%20of%20a%20switchable%20chimeric,pre%2Dclinical%20solid%20tumor%20model.

Human CD45 HI30 Pacific Blue BioLegend 304029 3:100
https://www.biolegend.com/en-us/products/pacific-blue-anti-human-cd45-antibody-3331?GroupID=BLG5926

Human CD45 HI30 PerCP-Cy5.5 BioLegend 304028 2:100
https://www.biolegend.com/fr-lu/products/percp-cyanine5-5-anti-human-cd45-antibody-4240

Human CD33 WM53 BV421 BD 562854 5:100
https://www.bdbiosciences.com/en-us/products/reagents/flow-cytometry-reagents/research-reagents/single-color-antibodies-ruo/bv421-mouse-anti-human-cd33.562854

Human CD33 AC104.3E3 PE-Vio770 Myltenyi 130-101-061 2:100
Discountinued

Human CD33 REA775 VioBlue Myltenyi 130-111-024 2:100
https://www.miltenyibiotec.com/US-en/products/cd33-antibody-anti-human-reafinity-rea775.html#gref

Human CD33 REA775 APC Myltenyi 130-111-020 2:100
https://www.miltenyibiotec.com/US-en/products/cd33-antibody-anti-human-reafinity-rea775.html#gref

Human CD34 AC136 APC Myltenyi 130-113-176 2:100
https://www.miltenyibiotec.com/US-en/products/cd34-antibody-anti-human-ac136.html#apc:30-tests-in-60-ul

Human CD34 AC136 PE-Vio770 Myltenyi 130-113-180 2:100
https://www.miltenyibiotec.com/US-en/products/cd34-antibody-anti-human-ac136.html#gref

Human CD34 AC136 PE Myltenyi 130-113-179 2:100
https://www.miltenyibiotec.com/US-en/products/cd34-antibody-anti-human-ac136.html#pe:30-tests-in-60-ul

Human CD34 AC136 VioBlue Myltenyi 130-113-182 2:100
https://www.miltenyibiotec.com/US-en/products/cd34-antibody-anti-human-ac136.html#vioblue:30-tests-in-60-ul

Human CD117 104D2 PE-Cy7 BioLegend 313212 5:100
https://www.biolegend.com/de-at/products/pe-cyanine7-anti-human-cd117-c-kit-antibody-4062

Human CD117 YB5.B8 PE BD 555714 5:100
https://www.bdbiosciences.com/en-us/products/reagents/flow-cytometry-reagents/research-reagents/single-color-antibodies-ruo/pe-mouse-anti-human-cd117.555714

Human CD271/NGFR ME20.4 APC-Fire750 BioLegend 345116 2:100
https://www.biolegend.com/en-us/products/apc-fire-750-anti-human-cd271-ngfr-antibody-16306?GroupID=GROUP28

Human CD271/NGFR REA844 APC Myltenyi 130-112-602 2:100
https://www.miltenyibiotec.com/US-en/products/cd271-lngfr-antibody-anti-human-reafinity-rea844.html?gclid=CjwKCAiAoL6eBhA3EiwAXDom5ty1zRUdvsLjjrKSqtK2P8Z1zpbDKjasVnlsOx1yx1J_MWaKOyRxEhoC5TUQAvD_BwE#apc:100-tests-in-200-ul

Human CD38 HB-7 BV510 BioLegend 356612 1:100
https://www.biolegend.com/fr-lu/products/brilliant-violet-510-anti-human-cd38-antibody-8530

Human CD38 HIT2 BV605 BioLegend 303532 2:100
https://www.biolegend.com/nl-nl/products/brilliant-violet-605-anti-human-cd38-antibody-8154

Human CD38 HB-7 PerCP-Cy5.5 BioLegend 356614 1:100
https://www.biolegend.com/en-us/search-results/percp-cyanine5-5-anti-human-cd38-antibody-8608?GroupID=BLG10099

Human CD3 UCHT1 PE BioLegend 300408 5:100
https://www.biolegend.com/en-us/products/pe-anti-human-cd3-antibody-865

Human CD3 SK7 FITC BioLegend 344804 2:100
https://www.biolegend.com/it-it/products/fitc-anti-human-cd3-antibody-6427

Human CD19 4G7 FITC BD 345776 3:100
https://www.bdbiosciences.com/en-eu/products/reagents/flow-cytometry-reagents/clinical-diagnostics/single-color-antibodies-asr-ivd-ce-ivd/cd19-fitc.345776

Human CD19 HIB19 BV421 BD 562440 2:100
https://www.bdbiosciences.com/en-us/products/reagents/flow-cytometry-reagents/research-reagents/single-color-antibodies-ruo/bv421-mouse-anti-human-cd19.562440

Human CD235a/GLYA REA175 FITC Myltenyi 130-177-688 2:100
https://www.miltenyibiotec.com/US-en/products/cd235a-glycophorin-a-antibody-anti-human-reafinity-rea175.html#gref

AnnexinV NA Pacific Blue BioLegend 640918 5:100
https://www.biolegend.com/en-us/products/pacific-blue-annexin-v-5476?GroupID=BLG6046

AnnexinV NA FITC BioLegend 640906 5:100
https://www.biolegend.com/en-gb/products/fitc-annexin-v-5161

Mouse CD45 30-F11 APC invitrogen 17-0451-82 1:100
https://www.thermofisher.com/antibody/product/CD45-Antibody-clone-30-F11-Monoclonal/17-0451-82

Mouse CD45 REA737 APC-Vio770 Myltenyi 130-110-662 2:100
https://www.miltenyibiotec.com/US-en/products/cd45-antibody-anti-mouse-reafinity-rea737.html#apc-vio-770:30-ug-in-200-ul

Mouse CD45.1 A20 BV650 BioLegend 110735 1:100
https://www.biolegend.com/en-ie/products/brilliant-violet-650-anti-mouse-cd45-1-antibody-7644

Mouse CD45.1 A20 PE BD 553776 1:100
https://www.bdbiosciences.com/en-us/products/reagents/flow-cytometry-reagents/research-reagents/single-color-antibodies-ruo/pe-mouse-anti-mouse-cd45-1.553776

Mouse CD45.1 A20 FITC BD 553775 1:100
https://www.bdbiosciences.com/en-eu/products/reagents/flow-cytometry-reagents/research-reagents/single-color-antibodies-ruo/fitc-mouse-anti-mouse-cd45-1.553775

Mouse Ki-67 B56 Alexa Fluor 647 BD 558615 2:100
https://www.bdbiosciences.com/en-us/products/reagents/flow-cytometry-reagents/research-reagents/single-color-antibodies-ruo/alexa-fluor-647-mouse-anti-ki-67.558615

Eukaryotic cell lines

Policy information about cell lines and Sex and Gender in Research

Cell line source(s)	HEK293T (ATCC)
Authentication	Cells were grown from a working cell bank obtained from ATCC and established in the laboratory for efficient lentiviral vector production. Cell line authentication was not performed.
Mycoplasma contamination	Test for mycoplasma contamination was negative.
Commonly misidentified lines (See ICLAC register)	No commonly misidentified cell lines were used.

Animals and other research organisms

Policy information about studies involving animals; ARRIVE guidelines recommended for reporting animal research, and Sex and Gender in Research

Laboratory animals	NSG (NOD.Cg-Prkdcscid Il2rgtm1Wjl/SzJ) and NSGW41 (NOD.Cg-Prkdcscid Il2rgtm1Wjl/SzJ KitW41/W41) female mice of 6-10 weeks of age were obtained from Charles River Laboratories (Calco, IT). Animals were maintained in Specific pathogen-free (SPF) animal research facilities with a 12h/12h dark/light cycle and standardized temperature (22 +/- 2°C) and humidity (55 +/- 5%).
Wild animals	This study did not involve wild animals
Reporting on sex	Only female mice were used for patient derived xenografts as they provide more robust human engraftment within peripheral blood and bone marrow. Sex of mice employed within the study does not affect the results of this study as murine cells were not analyzed.

Field-collected samples	This study did not involve samples collected from the field.
Ethics oversight	All experiments and procedures involving animals were performed with the approval of the Animal Care and Use Committee of the San Raffaele Hospital (IACUC: #807, #923, #1102) and authorized by the Italian Ministry of Health and local authorities accordingly to the Italian law.

Note that full information on the approval of the study protocol must also be provided in the manuscript.

Clinical data

Policy information about clinical studies

All manuscripts should comply with the ICMJEguidelines for publication of clinical research and a completedCONSORT checklist must be included with all submissions.

Clinical trial registration	N/A
Study protocol	N/A
Data collection	Primary AML patient samples from patients undergoing treatment at the Hematology and Bone Marrow Transplantation Unit of the San Raffaele Hospital were collected between 2005 and 2021 at the San Raffaele Hospital Hematology and Bone Marrow Transplantation Unit and processed at the San Raffaele Hospital Biobank laboratory (Milan, Italy).
Outcomes	N/A

Flow Cytometry

Plots

Confirm that:

- [x] The axis labels state the marker and fluorochrome used (e.g. CD4-FITC).
- [x] The axis scales are clearly visible. Include numbers along axes only for bottom left plot of group (a 'group' is an analysis of identical markers).
- [x] All plots are contour plots with outliers or pseudocolor plots.
- [x] A numerical value for number of cells or percentage (with statistics) is provided.

Methodology

Sample preparation	Freshly recovered or vitally frozen xenograft-derived bone marrow cells or frozen patient derived bone marrow mononuclear cells were thawed by drop addition of RPMI 20% FBS, washed, pelleted and resuspended in MACS buffer (Myltenyi, 130-091-221) or ice cold PBS+2%FBS. Cells were incubated 10 minutes with human or, for xenograft derived samples, human and mouse FcR blocking reagents (Miltenyi Biotec, #130-059-901, dilution 2:100, BD #553141, dilution 1:100) and then subsequently stained for 30 minutes at 4°C, washed, pellet and resuspended in PBS and sorted or analyzed immediately after.
Instrument	Immunophenotypic analysis were performed using one of the following instruments: BD FACSCanto II, BD LSRFortessa II, BD FACSymphony A5, Beckman Coulter Cytoflex S or Beckman Coulter Cytoflex LX. Cell sorting was performed on a BD FACSAria Fusion (BD Biosciences) using BDFACS Diva software.
Software	Data was collected on the BD FACSDiva software v.8.0.2 for FACSCanto II, LSRFortessa II FACSymphony A5. Data was collected on the CytExpert software v2.4.0.28 (Beckman Coutier) for Cytoflex S or LX. Flow cytometry data was analyzed with FCSExpress v6 or v7 (DeNovo software) and/or directly with R v4.0.3.
Cell population abundance	The purity of the sorted samples was determined by rerunning sorted samples on the flow cytometer.
Gating strategy	The gating strategy for each experiment is detailed in the methods section or Supplementary Figures of the manuscript.<br>Hierarchical gating was performed as follows:<br>Figure 3: Singlets (FSC-H vs FSC-A)/Physical parameters (FSC-A vs SSC-A)/ human cells (hCD45 vs mCD45)/ transduced cells (GFP vs mCherry or NGFR).<br>Supplementary Figure 1:<br>Strategy1: Singlets(FSC-H vs FSC-A)/Physical parameters (FSC-A vs SSC-A)/hCD45+ or hCD45low/Recovery of CD117+34- (Gate CD117+) & CD34+ cells.<br>Strategy2: Singlets(FSC-H vs FSC-A)/Physical parameters (FSC-A vs SSC-A)/Live/FITC- (CD3-CD19-CD235a-)/hCD45+ or hCD45low/ Recovery of CD34+ cells; cells not within CD34+ gate are furhter divided into recovery gates: CD117+ & inverse CD117+ gate (NOT-CD117+).<br>Strategy3: Singlets(FSC-H vs FSC-A)/Physical parameters (FSC-A vs SSC-A)/hCD45+ or hCD45low & FITC- (AnnexinV-CD3-CD19-CD235a-)/ Recovery of CD34+ cells; CD117+34- cells (Gate CD117+); and remaining CD34-117- cells (Gate P4).<br>Supplementary Figure 2:<br>Singlets (FSC-H vs FSC-A)/Physical parameters (FSC-A vs SSC-A)/ human transduced AML (hCD45+ & GFP+)/ GFPlow & GFPhigh.

- [x] Tick this box to confirm that a figure exemplifying the gating strategy is provided in the Supplementary Information.
