# Supplementary material for: Longitudinal single-cell profiling of chemotherapy response in acute myeloid leukemia
Source: Nat Commun. 2023 Mar 8;14:1285. doi: 10.1038/s41467-023-36969-0 (PMC9995364; doi:10.1038/s41467-023-36969-0)
Supplement: Supplementary file 4 — Description of Additional Supplementary Files [file 41467_2023_36969_MOESM4_ESM.docx]

**Description of Additional Supplementary Files**

Supplementary Data 1

Description: Single-cell RNA sequencing metrics of patient AML samples

Supplementary Data 2

Description: Marker genes for clusters identified in single-cell RNA sequencing of patient AML samples

Supplementary Data 3

Description: Differentially expressed genes in bulk RNA sequencing across comparisons of sorted GFP populations from treated or control patient derived xenografts

Supplementary Data 4

Description: Single-cell RNA sequencing metrics of patient derived xenograft samples

Supplementary Data 5

Description: Marker genes for clusters identified in single-cell RNA sequencing of patient derived xenograft samples

Supplementary Data 6

Description: Survival analysis input gene lists and model coefficients for large publicly available AML cohorts

Supplementary Data 7

Description: Table of reagents used for flow cytometry experiments
